# Using complexity theory to develop a student-directed interprofessional learning activity for 1220 healthcare students

**DOI:** 10.1186/s12909-016-0717-y

**Published:** 2016-08-08

**Authors:** Christine Jorm, Gillian Nisbet, Chris Roberts, Christopher Gordon, Stacey Gentilcore, Timothy F. Chen

**Affiliations:** 1 Sydney Medical School, University of Sydney, Edward Ford Building (A27), Sydney, 2006 Australia; 2 Faculty of Health Sciences, University of Sydney, Sydney, Australia; 3 Sydney Nursing School, University of Sydney, Sydney, Australia; 4 Faculty of Pharmacy, University of Sydney, Sydney, Australia

## Abstract

**Background:**

More and better interprofessional practice is predicated to be necessary to deliver good care to the patients of the future. However, universities struggle to create authentic learning activities that enable students to experience the dynamic interprofessional interactions common in healthcare and that can accommodate large interprofessional student cohorts. We investigated a large-scale mandatory interprofessional learning (IPL) activity for health professional students designed to promote social learning.

**Methods:**

A mixed methods research approach determined feasibility, acceptability and the extent to which student IPL outcomes were met. We developed an IPL activity founded in complexity theory to prepare students for future practice by engaging them in a self-directed (self-organised) learning activity with a diverse team, whose assessable products would be emergent creations. Complicated but authentic clinical cases (*n =* 12) were developed to challenge student teams (*n =* 5 or 6). Assessment consisted of a written management plan (academically marked) and a five-minute video (peer marked) designed to assess creative collaboration as well as provide evidence of integrated collective knowledge; the cohesive patient-centred management plan.

**Results:**

All students (including the disciplines of diagnostic radiology, exercise physiology, medicine, nursing, occupational therapy, pharmacy, physiotherapy and speech pathology), completed all tasks successfully. Of the 26 % of students who completed the evaluation survey, 70 % agreed or strongly agreed that the IPL activity was worthwhile, and 87 % agreed or strongly agreed that their case study was relevant. Thematic analysis found overarching themes of engagement and collaboration-in-action suggesting that the IPL activity enabled students to achieve the intended learning objectives. Students recognised the contribution of others and described negotiation, collaboration and creation of new collective knowledge after working together on the complicated patient case studies. The novel video assessment was challenging to many students and contextual issues limited engagement for some disciplines.

**Conclusions:**

We demonstrated the feasibility and acceptability of a large scale IPL activity where design of cases, format and assessment tasks was founded in complexity theory. This theoretically based design enabled students to achieve complex IPL outcomes relevant to future practice. Future research could establish the psychometric properties of assessments of student performance in large-scale IPL events.

**Electronic supplementary material:**

The online version of this article (doi:10.1186/s12909-016-0717-y) contains supplementary material, which is available to authorized users.

## Background

The importance of collaborative work in delivering quality care to patients or clients is widely accepted. However, there is equivocal evidence as to how health professional students should best be prepared for the challenges of a world in which safe and efficient care is delivered to a population that is aging, has high levels of chronic and complex disease and when technological and scientific advances abound. Future health professional working environments are likely to include more specialization within professions and the development of new kinds of healthcare workers. Practitioners will need to ‘negotiate meaning, resolve epistemological differences, develop shared understanding and communicate … to a broad audience’ [1]. This will take place increasingly often and with people they may not know.

Students need interprofessional learning (IPL) that prepares them for interprofessional practice (IPP). While interprofessional education (IPE) and IPL are used somewhat interchangeably in the literature, we prefer IPL as it emphasizes the active process of learning. We use Freeth et al.’s definition of IPL: ‘learning arising from interaction between members (or students) of two or more professions’ ([2] p18). In healthcare, ‘teams are most often ad hoc and may change on a weekly, daily, or even hourly basis for any given patient’ ([3] p67). Thus Edmondson’s concept of ‘teaming’ [4] is highly relevant to IPL. Teaming refers to the behaviours necessary for successful function in dynamic, ad-hoc teams: speaking up, collaboration, experimentation and reflection [5]. Influenced by Bleakley [6] and considering the contested and highly political discourse on teams and teamwork in healthcare, for this paper, we define teamwork as the process of conscious collaboration.

In preparing students for the workforce, universities have struggled to create and maintain authentic IPL activities that are inclusive for whole cohorts. Separate faculties often make shared work difficult [7] and sustainability has proven challenging. Resource intensive extra-curricular activities, involving a small proportion of the cohort are common. [8–13] Several groups have responded to the scheduling difficulties of IPL by investment in e-learning. For example, Bournemouth university developed a complicated virtual community with evolving scenarios as triggers for problem based learning activities [14] but students evaluated the learning activity poorly. Another project educating 2,800 students each year via the use of on-line discussion groups had disappointingly poor results [15]. Some doubt whether the evidence for the benefits of IPL is sufficient to justify the effort of implementation and diversion of discipline specific resources [12]. The practical difficulties of providing IPL have resulted in limited opportunity to develop a strong evidence base [11, 16–22] including lack of attention to relevant educational theory until quite recently [18, 22–24].

### Theoretical principles underpinning the educational design

Health professional education has in the past been dominated by a pre-occupation with reliable measurement of the skills of individual students, thus ignoring what ten Cate et al., describe as ‘the kinds of higher-order thinking and acting that constitute competence in demanding work’ [25]. Important parts of the work students will do in the clinical workplace cannot easily be codified and are not an individual enterprise, for example recent enquiry revealing the intensely social nature of infection control practice [26]. Thus, in addition to traditional behavioural and learning theories [27], a range of other theories from psychology, social science and business have now been promoted for use in IPL. [24, 28] These are diverse, including for instance, the use of role theory to analyze gaps in the IPL literature [29], activity [30] and social capital theories [31]. The range available was described in 2009 by Hean et al., as ‘an un-navigable quagmire’ [27].

For our large scale IPL event complexity theory proved a good fit. We used it as our overarching theoretical basis for both the design of the learning activity and as a framework for evaluation. In education, complexity theory is one of a number of socio-material learning theories which de-emphasise the individual and focus on the dynamic social nature of learning. That is, it develops from and is inseparable with, social and material relationships (the latter pertaining to elements such as environment, artifacts, technologies) [32, 33]. Complexity theory has origins in economics and biology (and further back in the mathematics of chaos) and there are different definitions and elaborations associated with its application in particular fields. It is an approach to studying complex systems which is not linear or reductionist but focuses on the interactions between system components ‘as the foundation from which the properties of a system emerge’ [34] and accepts unpredictability of outcomes. Healthcare itself can be understood as a complex adaptive system at many levels [34] and complexity theory has special relevance to IPP. This is because of its relationship focus [34], its emphasis on adaptation and collective learning in teams [24] and sensitivity to the difficulties of everyday collaborative practice [24, 29]. IPP involves diverse perspectives and requires the construction of 'common ground', conflict negotiation and synthesis [30, 35].

Cooper et al suggest that complexity can enable IPL to ‘escape from the tradition of a linear paradigm for what is essentially a new way of learning’ [36]. There is as yet, scant literature applying complexity theory to *design* of specific educational interventions in health professional education [37, 38]. The focus has been mainly at course level, not at the level of detail that has been described for instance for game design [39] and physical education [40].

When complexity theory is applied to collaborative professional practice, four concepts in particular are emphasised: emergence, diversity, self-organisation and nested systems. [35] In this research we focus on the first three concepts. Some theorists consider diversity the most important *resource* of a complex system [33] but emergence is a central concept in education [35], where from multiple interactions between students emerges dynamic structures that exceed their parts. Emergent products have a nature that cannot be specified fully in advance and can be considered the ultimate product of effective IPL and practice (they can include diagnoses, care plans and research [33]). McMurty considers that a diverse and functional team creates:

*‘learning … beyond the sum of the individual professionals’ contributions. … new collective knowledge that not only exceeds their individual understandings but represents knowledge that could not have been predicted in advance of their collaboration’* [33].

In this research the emergent products are considered to be the students’ collective knowledge, which is articulated in their assessment. Our overarching research aim was to explore the extent to which the IPL activity prepared students for future practice by engaging in a self-directed (self-organised) learning activity founded in diversity (including students from different disciplines, and as always in group work, different abilities) where their assessable products would be emergent creations.

### Research questions

Our specific research questions were:

1. Is it feasible to deliver a large scale (*n*= > 1000) student-directed case-based IPL learning activity including assessment of emergent products as an embedded component of existing curricula?
2. To what extent was the IPL activity acceptable to participating students?
3. What is the evidence that students achieved IPL learning outcomes from the perspective of complexity theory?

## Methods

### Complexity theory based educational design

The educational design was underpinned by the three key components of complexity theory; diversity, self-organization and emergence. First, the principle of diversity was incorporated in two ways, by including a range of disciplines in every team and by case development undertaken with the intention of maximizing display of student diversity. That is, we developed complicated patient cases which required more than simple application of each student’s discipline-specific knowledge. This countered students’ wishes to be competent only in their own profession [37]. It ensured they engaged with IPL because of perceived relevance providing a context for meaningful interaction [32]. Our 12 complicated cases were designed to achieve this (a sample case, terminally ill ‘Jane Murphy’, is provided in Additional file 1).

Second, a social learning process was encouraged to enable students to produce new collective knowledge through self-organisation and negotiation. Students needed to ‘elicit, build on and challenge one another’s ideas’ [36] to create the integrated patient-centered management plan.

Third, the notion of emergent products in complexity theory guided the design of the summative assessment. Students had to submit a one-page evidence based management plan and a five-minute video that illustrated their patient case and their collaborative team-based approach to the patient’s care. Video is a novel assessment modality for health professional students and one that encourages creativity. It is suggested that group educational tasks lend themselves to a complexity based pedagogy if there are practices that promote ‘self-organization, adaptation and creativity’ [40]. Producing a video provides such a practice (including scripting, acting, devising camera angles, creation of visual and sound effects etc.) facilitating novel presentation of case management.

The challenging clinical nature of the cases and the limited time allowed meant ‘teaming’ (specifically co-operation, negotiation and task allocation) was essential to complete these assessment tasks. We suggest that just as in interprofessional simulation, ‘a simple scenario merely requires co-operation’ [41] hence our quest in the case design was to include complications that necessitated collaboration and increased the likelihood of emergence. Marking rubrics were designed to promote emergence by valuing both diverse thinking (creativity) and evidence of integrated discipline knowledge (a cohesive patient-centred plan) (rubrics are provided in Tables 1 and 2).

**Table 1:** Video Assessment Rubric

	Poor 1	Satisfactory 2	Good 3	Excellent 4
Patient issues	Issues faced by the patient and family not evident.	Describes the major issues faced by the patient and family.	Depicts appreciation of depth and/or breadth of issues faced by the patient and family.	Depicts considerable appreciation of depth and breadth of issues faced by the patient and family.
Interprofessional negotiation	Does not display negotiation, shared goal setting and shared decision making.	Shows limited appreciation of negotiation, shared goal setting and shared decision making.	Shows appreciation of negotiation, shared goal setting and shared decision making.	Sophisticated approach to negotiation, shared goal setting and shared decision making.
Interprofessional management plan in action	Management plan not evident in video.	Video depicts limited evidence of management plan in action.	Video depicts good evidence of management plan in action.	Strong depiction of co-ordinated and well executed interprofessional care.
Effective use of video medium to engage audience	Poor use of video medium.	Appropriate use of video medium.	Engaging.	Highly engaging and memorable.

**Table 2:** Abstract Assessment Rubric

	Poor 1	Satisfactory 2	Good 3	Excellent 4
Identifies and prioritises issues	Displays minimal awareness of the priority issues for the patient and family.	Superficially identifies the priority issues for the patient and family.	Identifies and justifies the priority issues for the patient and family.	Comprehensively identifies and justifies the priority issues for the patient and family.
Management plan contains specific strategies to address issues	Recommends few specific strategies to address some of the issues.	Recommends specific strategies to address some of the issues.	Recommends specific strategies to address most issues.	Recommends a comprehensive range of specific strategies to address all issues.
Includes evidence for management plan	Minimal evidence for management plan.	Some evidence for management plan.	Solid evidence for management plan.	Comprehensive and up to date evidence for management plan.
Communication of management plan	Management plan is disjointed, poorly organized and poorly written. It appears to be stitched together from various materials.	Management plan is coherent in parts and somewhat organized. Some aspects well written.	Management plan is coherent, clear and well organized. Well written.	Management plan is highly coherent and integrated. Well written.
Global assessment of abstract	Poor	Satisfactory	Good	Excellent

Finally, peer marking (of videos produced by other teams) assisted students in developing evaluative judgement of their team performance compared to that of other teams. Green notes that when ‘students review the work of their peers, they invariably reflect back on their own work and consider ways of improving it…[as] before reviewing the work of peers, students will have already spent considerable time producing work in the same domain topic themselves’ [42].

### Logistics for the IPL activity

The IPL activity, which we named the Health Collaboration Challenge (HCC), was conducted at a major metropolitan Australian university, which has over 12 health professional degree programs. There was no prior shared IPL strategy in place, and existing IPL activities were small scale without reliable cohort coverage. Project funding as well as faculty and Unit of Study (UOS) co-ordinator support allowed this activity to be embedded as a mandatory assessed component within the curriculum of a selection of eight degree programs. We were required to negotiate the mandatory assessment grading with each UOS co-ordinator, thus IPL assessment contributed to student assessment as either satisfactory/unsatisfactory or a weighted grade depending on the program specific requirements.

The IPL activity comprised of three major components: 1. A discipline-based face-to-face briefing session followed by individual student pre-work; 2. Central interprofessional learning experience, with a face-to-face briefing session prior to face-to-face team work; 3. Faculty assessment of written management plans and peer assessment of videos.

The intended IPL learning outcomes for students were to:

- Understand the contribution of a range of different health professions to meet complex patient care needs
- Integrate and prioritise key contributions from different health professions into a patient management plan
- Evidence the application of a collaborative approach to problem solving with different health professions for a challenging creative task

The HCC was adapted from a previously piloted volunteer program [43] which was developed from work first created in Canada [44] and now formularised [45]. A total of 1220 pre-registration students from eight disciplines took part in the IPL activity (Table 3).

**Table 3:** Participant disciplines and year of study

Discipline	Number of students	Cohort and Program
Medicine (M)	309	1^st^ year of 4 years G (graduate entry)
Nursing (N)	301	3^rd^ year – 3 years UG (undergraduate) & 2^nd^ year - 2 years GEM (Graduate Entry Masters)
Pharmacy (P)	233	4^th^ year – 4 years UG & 2^nd^ year – 2 years GEM
Speech Pathology (SP)^a^	135	2^nd^ year – 2 years GEM & 3^rd^ year – 4 years UG
Diagnostic Radiography (DR)^a^	73	2^nd^ year – 2 years GEM
Occupational Therapy (OT)^a^	73	2^nd^ year – 2 years GEM
Physiotherapy (PT)^a^	70	3^rd^ year – 4 years UG
Exercise Physiology (EP)^a^	26	2^nd^ Year – 2 years GEM

^a^Referred to as ‘Health Sciences’ disciplines

Student teams were provided with a challenging patient case study, which necessitated an interprofessional approach. Case studies were scripted by clinicians and based on real patient cases and then modified by the research team. Discipline experts and UOS co-ordinators reviewed the cases to ensure appropriateness. Cases included in-hospital and community care, all required input from multiple health professions (e.g. Jane Murphy, Additional file 1). There were 12 cases in total with each case being allocated to between 14 and 19 student teams. Students were assigned to teams of 5-6 members, making 208 teams. All teams had at least one medical, nursing and pharmacy student together with combinations of other health disciplines.

The individual on-line pre-work required students to read their case study and consider disciplinary aspects relevant to the case. This was provided via the Learning Management System (LMS) (Blackboard) one week prior to the face-to-face event (together with a team number and a list of team members).

For the central IPL experience we brought together approximately 400 students on three different days (September 2015) in a large sporting stadium (approximately 1280 m^2^). To manage the timetabling incompatibility across disciplines, a single day was cleared in the schedules of all students to create this opportunity. Some disciplines were spread across all three days, while other disciplines (e.g. exercise physiology and diagnostic radiology) all attended on a single day. Day allocations were made to suit each UOS and enable a good mix of disciplines in every team.

Upon arrival, students were directed into the tiered stadium seating and a 10-min briefing by members of the research team was used to encourage students to participate genuinely and to provide an opportunity for questions to be answered. Students then gathered in their teams at labelled areas through the stadium and were given a package of four team-building problem-solving exercises (written and geometric puzzles). After completion (approximately 20 mins), teams submitted their answers to a staff member along with a registration sheet recording attendance.

**Fig. 1:**
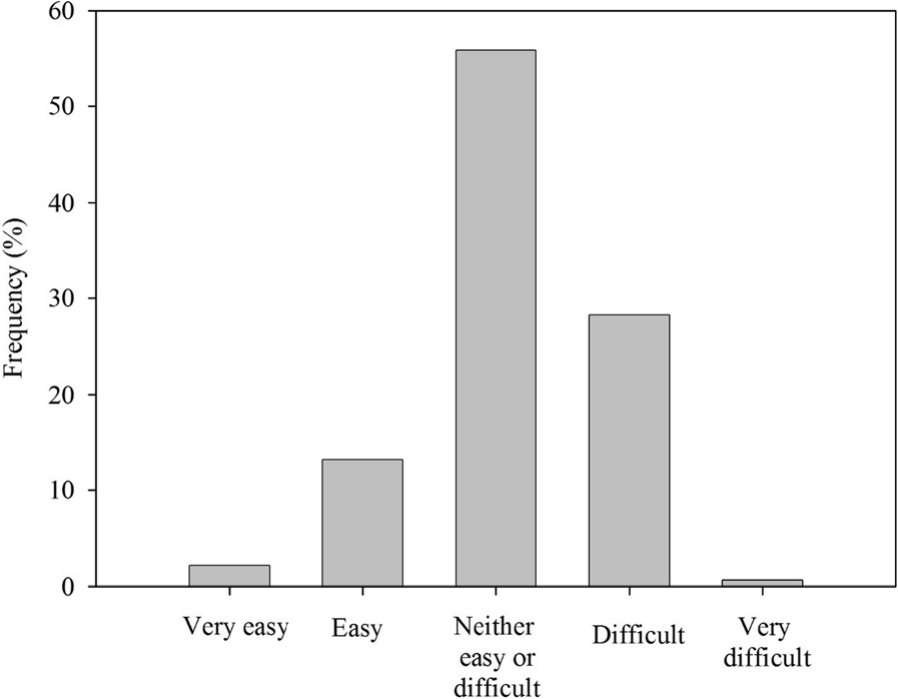
HCC participants’ ratings of the degree of difficulty of the case study

**Fig. 2:**
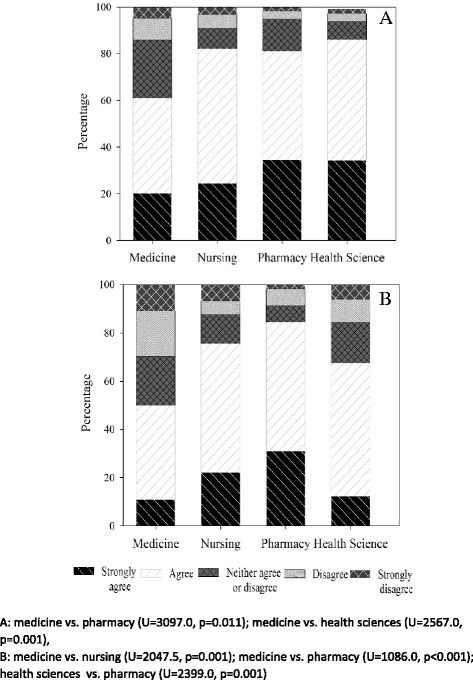
Medicine, nursing, pharmacy and health science student ratings about the relevance of the case study to each student (**a**), and overall the HCC was a useful learning activity (**b**)

Student teams then left the stadium and worked independent of academic staff for the rest of the day to produce a 5-min video for their case study and to complete a one-page patient management plan which provided evidence for interventions included in the video and details of an interprofessional framework that underpinned the team’s plan. Video and management plan assessment rubrics, which had been validated by the research team, were provided to students (Figs. 1 and 2). Students were encouraged to use their own technology (smart phones, tablets etc.) to film and edit their video. Hints and tips for shooting and editing videos on mobile devices were provided on the LMS. Exemplar videos from the previous year’s pilot were also available for students to view. The student teams had 48 h (from the stadium event) to complete their video and abstract and upload them to the LMS.

The third component of the IPL activity required students, as individuals, to peer mark two videos from two teams who had completed the same case. This was to be completed within 42 h of release of the peers’ videos and was coordinated using the LMS.

The management plans were marked separately by the five academic members of the research team who were blinded to the student team videos. Approximately four weeks after completing the IPL activity, each team was emailed their results: their average score (max score = 4) for each of the four video assessment rubric criteria, a global result for their abstract (poor/satisfactory/good/excellent) and a paragraph of generic feedback (dealing with specific clinical and teamwork issues that the students had handled well or neglected etc.) from the abstract marker for each patient case.

### Data collection

Students were invited formally to participate in research, including access to their assessment data. They were able to opt out, as standard, and many did not complete the post-event evaluation. Research question 1 (feasibility) was addressed by collecting a record of student attendance at the stadium briefing; assessment completion rate; number of late assessment submissions; and number and type of technical issues experienced by students and e-learning staff. It was also addressed through a researcher-designed post-learning activity survey which contained close-ended questions adapted from The University’s course evaluation survey and additional items specific to the IPL activity. We also included three open ended free text questions: ‘What aspect/s of your team's patient case study worked well? What aspect/s of the patient case study did your team find challenging? Is there anything else you would like to tell us?’. The SurveyMonkey^TM^ link to the questionnaire was provided to students in the results dissemination email. Research question 2 (acceptability) was also addressed by this questionnaire and research question 3 (specific IPL learning outcomes) assessed through the student performance as reflected by their assessment marks (for the video and abstract) and via analysis of their free text evaluation comments.

### Data analysis

A mixed methods approach [46] was used to answer our specific research questions. This approach enabled us to develop a more complete understanding of our research questions and to examine processes along with outcomes [47]. Student attendance rates and student assessment completion data were analysed descriptively. A log of technical issues was collated. Descriptive statistics were computed for the post-HCC evaluation questionnaire using SPSS Statistics^TM^ (version 22). Data were assessed for normality using the Kolmogorov-Smirnov test. Kruskal-Wallis H test was used to determine if differences existed in the evaluation questions between student health professional groups (health science, medicine, nursing and pharmacy). When significance was found, *post hoc* pairwise comparisons were analysed using Mann-Whitney test and corrected for using Bonferroni method. Alpha was set at 5 %.

Qualitative free text data from the questionnaire was analysed thematically [42]. Two researchers experienced in qualitative research (CJ and GN) undertook the initial analysis. De-identified text data was read independently by both researchers to familiarize themselves with the data. Each researcher assigned codes to the free text independently to reflect units of meaning and codes were grouped and collapsed into larger codes. At this stage complexity theory was not used to guide the coding, which was inductive in nature, although the authors were familiar with this theory. Next, the researchers met to compare and contrast coding, clarify and negotiate variations in coding and understandings. A third researcher (CR) independently assessed thematic clustering. At a further meeting the authors discussed the value of using complexity theory as the conceptual framework for this paper, and subsequently developed a thematic framework informed by complexity theory over further meetings. This framework was applied to the dataset by CJ and GN to establish trustworthiness, and checked for new and emerging issues of importance that would extend the analysis. An Excel spreadsheet (Microsoft^TM^) was used to manage data.

## Results

Of the 1220 students who participated in the HCC, 328 (26.24 %) chose to complete the program evaluation survey with 605 free text comments being submitted.

### Was this large scale IPL activity feasible?

All 1220 students successfully completed all required components of the IPL activity, including video and management plan submission and peer video assessment. Only one student failed to attend without making contact. This student was followed up and attended the Saturday supplementary event (to accommodate students who were ill, had family or religious observance on their allocated day etc.). Less than a dozen teams had issues uploading their video. Some had not followed the instructions provided and some did not have access to a wired internet connection at the time they tried to submit. Only three teams submitted after the submission deadline, but only by two hours.

To report against feasibility, we also analysed this learning activity against requirements suggested necessary for delivery of a successful and sustainable IPL curriculum (Table 4).

**Table 4:** Elements required for successful and sustainable IPL curriculum versus HCC experience

Elements suggested as essential for an IPL program	Analysis of HCC experience
Long term dedicated support and budget [48].	University grant funded initially; and for a subsequent 1-2 years but HCC relatively low cost because student-directed.
IPL performance metrics [49]. Rigorous assessment so that the evidence that IPL works is provided [50].)	Included- a large scale student-directed exercise enables and requires substantial data collection and analysis.
Faculty development program/training (e.g. in small group IPL facilitation) [51].	Not needed – a skeleton academic team of five developed and ran the HCC.
An academic calendar that allows for IPL. [48, 52]	Minimum shared scheduling required – a single student day.
Teaching spaces for small group work [37].	Made available to 45 % of students, most used public campus learning spaces.
Required participation of healthcare programs [53], i.e. IPL is mandatory [55].	The HCC is suitable for mandatory participation
Development of a formal IPL department or organizational home [56]. Secure governance arrangements are a priority.	Will be needed to provide long term support and development
Links between higher education and health [58].	Minimum links are needed – primarily to source authentic case studies

### To what extent was the learning activity acceptable to students?

Overall, the complexity of case studies was appropriate, with the majority of the 328 students who completed the evaluation rating them as neutral (55.8 %) or difficult (28.2 %) (Fig. 1). All videos and abstracts (from the whole cohort - 208 teams) were marked as satisfactory or above.

The majority of the students who completed the evaluation (70 %) agreed or strongly agreed that the HCC was a worthwhile learning activity and 87 % agreed or strongly agreed that their case study was relevant. Use of video as an assessment task was the least positively rated aspect of the HCC, with less than 50 % of respondents agreeing or strongly agreeing that it was useful.

**Fig. 3:**
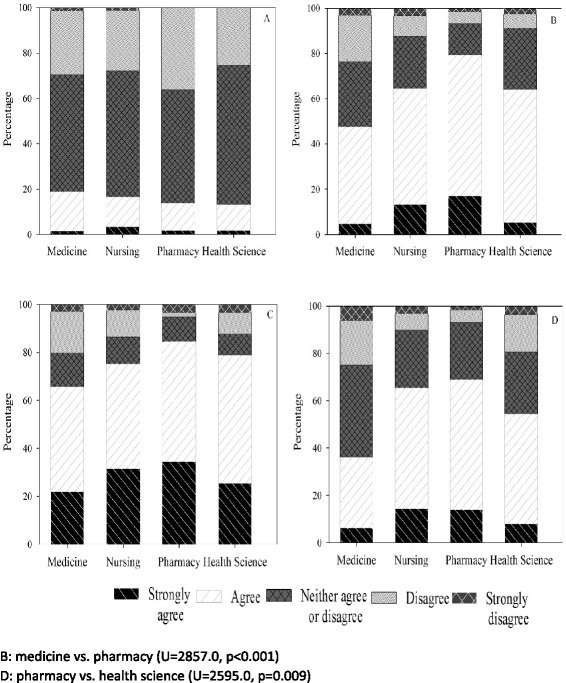
Medicine, nursing, pharmacy and health science student ratings of: development of problem solving ability (**a**), sharpened analytic skills (**b**), development of teamwork skills (**c**), and confidence related to tackling unfamiliar challenges (**d**) following the HCC

**Fig. 4:**
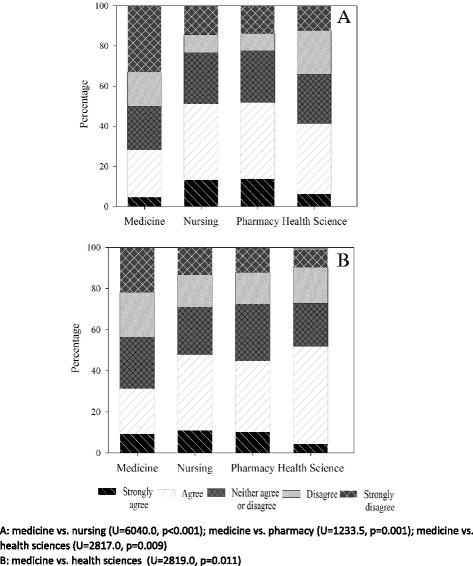
Medicine, nursing, pharmacy and health science student ratings of: making the video was a useful teamwork task (**a**), and peer video-marking was a useful learning activity (**b**) during the HCC

Kolmogorov-Smirnov test revealed statistical significance across all evaluation questions (all *p <* 0.05). As data were non-normal, Kruskal-Wallis test was used to test for differences between the student health professional groups. Overall, students highly rated the HCC with the Kruskal-Wallis H test revealing statistical differences between health professional students (χ^2^(3) =22.85, *p <* 0.001). More nursing and health science students reported that the case studies and HCC were useful learning activities than medical students (both *p <* 0.05; Fig. 2). Moreover, pharmacy students rated the HCC as having a greater impact on their analytical skills than medical students did (all *p <* 0.05; Fig. 3). Similarly, nursing, pharmacy and health sciences students reported that making the video was a more useful teamwork task and learning activity than did medical students (all *p <* 0.05; Fig. 4). Note, *post hoc* analyses comparing differences between health professional student groups (Mann Whitney with Bonferroni correction) are shown in Figs. 2, 3, and 4.

Thematic analysis, of the free text data contributed by the evaluation survey respondents, informed by complexity theory resulted in two overarching themes: student engagement and collaboration-in-action.

### Theme 1: engagement with the IPL Activity

Engagement is central to the dynamic social approach to learning that is described by sociomaterial theories [32, 33]. There were barriers and enablers to student engagement. The main engagement subthemes dealt with the complicated case study, the curriculum context, the video task and the dynamic social nature of this novel activity.

### Complicated case study

The complicated case study content was an enabler for team engagement. The cases were considered authentic for professional practice:

*M38 ‘realistic, something that we all will come across in our careers’*

At the simplest level, working through the cases enabled students to gain specific new clinical knowledge from other students (e.g. about dangerous drug interactions) and facilitated learning about the work of other professional groups. Some students suggested that didactic pre-education about what the professions did would have been useful, a reflection of the lack of prior IPL activities in their disciplinary degree programs. Students perceived that the meaningful work the case discussions required from team members were a large contributing factor in meeting the intended learning outcomes of the event:

*SP4 ‘Inclusion of roles and input for each team member which promoted learning about each discipline's background, their responsibilities and rationale for what they would do’*

The absence of health professions that were considered key to the case study was mentioned by some. While students were in general, allocated appropriately to cases designed for their disciplines, there were a few deficiencies in team membership and some respondents requested input from social work and psychology students (not participating in HCC).

Students identified that the complicated cases engendered and required collaboration, creating a satisfying learning experience:

*M7 ‘the case was quite complex, so lent itself well to an interdisciplinary approach’*

*P39 ‘making each member imperative in working out our case’*

Collaboration in student teams thus appeared to be an important precursor to the creation of emergent products, in this case producing the two assessment items as a team.

### Impact of the learning context

Whilst the HCC appeared to support student engagement, there were three potentially remediable contextual factors that were a barrier to student engagement and impaired the value of this required learning activity for participants. First, the variations in the summative assessment policy for differing degree programs was considered ‘unfair’. Some students perceived they needed to just attend to gain satisfactory completion where others felt pressured to achieve good results in the HCC assessments for credit in their program.

*P52 ‘Recommend weighting of assignment to be equal for all disciplines. This requires all students to try their hardest to achieve more than just a “pass” mark’*

Second, for other students timing was poor (close to other assessments) or took them away from clinical placement, leading some to conclude that the exercise was a ‘waste of time’ in these circumstances. Others overcame these challenges and were determined to engage:

*SP17 ‘Although it was a stressful day, given that I had a lot of other assessments on at the same time, I really enjoyed it! There were a lot of laughs, and I really enjoyed getting to know how other allied health professionals work in a safe environment. Only until it was over did I realise how much I enjoyed it. I wish I could do it again when it wasn't crunch time at uni!’*

Third, there was discussion about the equivalence of experience of the students from each of the participating disciplines. For example, it was felt that the inclusion of first year medical students (a medical school decision based on other curriculum demands) was problematic:

*P2 ‘I think it would have been better if all the students were at a similar stage in their degree. Whilst pharmacists, nursing and radiography were all in their final year the medical students were only in first year. It would be great to work with medical students who were a little further into their degree.’*

The diversity of the student groups challenged the typically stereotypical view of medical students as clinical leaders of teams and highlighted issues around leadership and power that are frequently contentious in interprofessional groups. Some medical students appeared self-conscious or humbled by this circumstance:

*M1 ‘I really appreciated the opportunity to meet students from other disciplines, although I felt under-qualified due to the fact that I was in first year whereas the nurses and physio had a very impressive clinical knowledge base and much more experience.’*

Involving junior medical students added tension to the learning experience of some teams as some of the medical students expected to lead and did so regardless of their junior status:

*N7 ‘The medicine students could tone down the sass. Please and thank you.’*

*P37 ‘The doctors took on their role of managing the bulk of patient care very enthusiastically even though they were not equipped with the skills to do so just yet. This made things very slow going.’*

### Video task

The majority of students engaged with the assessment plan, and produced the videos with a varying degree of success according to the averaged marks of their peers. Less than a handful of videos were deemed to be of poor. Some students made explicitly positive comments about the video task:

*P39 ‘The video aspect allowed us to explore our creativity and my group had a lot of fun in particular when filming our video.’*

However, many students complained about the requirement to make a video, considering the task difficult and not relevant to their training.

*N48 ‘Making it a video task felt time-consuming, unnecessary and inappropriate for the task …[and] the biggest challenge was troubleshooting software problems and problems with filming devices.’*

Some of the students who found the technical aspect of the video task off-putting otherwise found valuable learning in the case discussion. One of the students demonstrated no sense of irony in suggesting that healthcare science was not amenable to creativity:

*M26 ‘I want to be honest and say I did not enjoy the video task and neither did my team. The case was good and working together was great, I felt like it was worthwhile doing the abstract and I did learn something. But getting a bunch of predominantly science students to do a creative project like a video is borderline painful.’*

There is a contradiction inherent in a comment such as: ‘I personally really enjoyed the teamwork aspects but not the task itself’ (DR11). The task may have created/necessitated the teamwork. The above student comments (M26 & P39) also illustrate the dichotomous views espoused about creativity in the context of professional health education. Some students pointed to the decisions about prioritising creativity inherent in deliberating about video content, listing a challenge as:

*P43 ‘Figuring out if we should focus more on creativity and engaging the audience, or the clinical and nitty gritty details to help Jo [their patient case].’*

### Dynamic social learning

This team based learning activity was described by many as fun or as ‘an exciting experience’ *(SP14)* which took students out of their comfort zone, and brought fresh perspectives to their learning.

*DR2* ‘*[I] really enjoyed getting out of the [Health Sciences disciplines] campus and meeting other students of different disciplines - would do it again.’*

In part because of lack of prior IPL experience, students had been socialised into believing that IPL events would not be a source of valuable learning. This was mitigated by the educational design of the HCC (including constructively aligned assessment).

*N74 ‘I wasn't very optimistic about the whole activity in the beginning but by the end of this activity I found that I very much enjoyed this opportunity to work collaboratively with other students. It was quite different from any other group work I have ever done. I believe that I gained more out of this collaborative group work than any other group assignment that I have undertaken.’*

### Theme 2 – collaboration-in-action

While the first theme encompasses factors that energised student engagement in a dynamic IPL activity, the second theme spot lights student experience of the *process* of collaboration that was necessary to create their emergent assessment products. Student experience of collaboration-in-action was reflected in subthemes of ‘communication’, ‘negotiation and conflict resolution’ and ‘integration and emergence’.

### Communication

Students focused on this fundamental process aspect of collaboration in various ways, sometimes with mention of listening or ‘great discussion between the different disciplines…’ *OT20.* The complicated cases were again suggested by students as being important for stimulating or requiring discussion:

*M64 ‘Our group dynamic was good with each of the disciplines contributing meaningfully. The case was quite complex, meaning a lot of discussion was required.’*

Some students also mentioned respectful and supportive aspects of communication: ‘I enjoyed shared decision-making and felt respected in the group’ (P38). While another described: ‘patience amongst the group… Ideas and helpful critiques given in a caring way’ (SP17). The notion of critiques being given in a ‘caring’ way is interesting. Extending the earlier results describing the contribution that the complicated case study made to engagement, it seems possible that the ‘empathy-inducing’ bio-social complexity of the cases resulted in a framing effect for some students’ group behaviour. The quote below hints at this:

*N64 ‘We had a good team that worked well, listened to each other and all contributed. We thought holistically and tried to include as much psychosocial and person-centred care as possible.’*

### Negotiation and conflict resolution

IPP requires the construction of 'common ground' and conflict negotiation prior to synthesis [30, 35]. Negotiation was primarily around team member roles, necessary to create the fundamental common ground for case management:

*OT14 ‘Areas that could be addressed by multiple professions- this was challenging at first but prompted conversation among team members to explain their reasoning and negotiate the best option for a care plan.’*

The negotiation required to create an integrated patient centred management plan stretched students, but was feasible for most teams. Working with paper (static) cases potentially added more negotiation challenge to the creation of an integrated holistic management plan, due to the inability of the team to ask and confirm patient and carer preferences. Interestingly, some of the senior speech pathology students, who had considerable clinical teamwork experience, had sophisticated insight into the learning interprofessional negotiation engendered:

*SP51 ‘Team members were able to identify and resolve professional cross-over issues clearly and efficiently, providing each individual with heightened insights into specific assessment and treatment considerations.’*

Conflict resolution was discussed by some as a normalised part of the experience - their discussion of ‘conflict’ being quite affect free:

*M52 ‘[Challenges included] working out how to treat Jane, and working out how to work together e.g. one of the students began by telling everyone what to do but we renegotiated those roles to bring forward others' voices in the video.’*

*SP50 ‘The time constraints did not allow my team much time to build rapport and as a result there were some conflicts between members that needed to be resolved during the preparation and planning stages.’*

For others, conflict proved difficult to resolve and they were thus not able to manage effective collaboration. For instance, some depicted difficulties in priority setting as a complaint about others:

*M22 ‘People making their discipline the centre of the management plan, and being unwilling to budge.’*

A few groups were dysfunctional and students’ descriptions of these are affect laden:

*M43 ‘After hours of disagreement, we all decided that we wanted to be able to leave the uni grounds before 6 pm. We sort of banded together to produce the video…. I had a group member who was incredibly disparaging, using words like “that is just stupid and other assorted insults” and refused to listen to any input.’*

### Integration and emergence

This final subtheme relates to the creation of their emergent assessment products, the video and the case management plan. Our theoretical stance considers emergence as the ultimate outcome of effective IPL and IPP [33]. Students described how they were able to consider the priorities of others, again part of construction of ‘common ground’:

*N48 ‘It was useful to understand healthcare from the perspective of other disciplines. It was interesting to see how priorities and treatment of health problems differed between disciplines, especially medicine and nursing.’*

*P37 ‘It was interesting to hear the perspective the doctors and nurses offered on the patient that I would have otherwise not considered.’*

Students’ completed assessment products were cohesive yet highly varied in approach (unpublished data), thus *demonstrating* emergence and reflecting the meshing of individual discipline priorities into the patient-centred plan. In their evaluation responses some students explicitly *described* the process of collaboration to create or synthesise new knowledge. That is, the process of building from team members’ discipline-specific expertise to create the collective knowledge embodied in the patient-centred case management plan (and displayed in the two assessment products):

*OT2 ‘Discussing as a group and then branching off into each of our expertise to tackle certain points, and then going through it as a team to put everything together.’*

*SP36 ‘Everyone was able to contribute, and we worked well to synthesise ideas from different disciplines in a way that would still keep the interests of the client as the core focus.’*

## Discussion

We have demonstrated the feasibility and acceptability of a mandatory large-scale student-directed interprofessional learning activity (research questions one and two). Low fidelity simulation exercises such as group work to prepare a case management plan have been successful in other settings [48–50]. One university has reported on a single 900 student session of 180 min that included work in small groups [51]. However, tutor requirements can be formidable [48, 52] (e.g. 1:6 tutor-student ratio). At the University of Alberta, 1000 interdisciplinary health students undertake activities guided by 50 trained facilitators, with pairs responsible for a classroom of 50 students (six teams) who work through case scenarios [37].

Our IPL activity was designed to overcome barriers to scalability and sustainability and provide a prototype for a future university-wide approach to interprofessional activities also suitable for non-health faculties. The student-directed nature and the use of peer-marking reduced resourcing requirements. (Interestingly, the students made no free text comment on their experience of peer marking process). We demonstrated the feasibility of embedding a new activity in multiple disciplinary UOS and within existing teaching workloads (with only a small guiding academic team, but with dedicated funded administrative support). Many elaborate attempts at IPL have produced disappointing returns on the educational investment made and have been difficult to sustain, thus recent evaluation recommendations include prioritisation of description of context [53]. It is also suggested by Fraser and Greenhalgh that attention to process is ‘the distinguishing characteristic of productive non-linear learning’ [54]. Hence we have provided detailed description of both process and context together with an analysis against curriculum elements suggested as essential for IPL by others (and this was favorable).

This learning activity was demonstrated by quantitative and qualitative data to be acceptable to students. Student satisfaction is particularly important in IPL as it has been suggested that ‘early experiences … may even cause harm if students believe they are practising as part of an interprofessional team when in fact the ‘team’ is dysfunctional and the activity lacks the potential benefits students have been promised’ [55]. Student feedback was extremely positive for an activity that was an example of an ‘ad hoc and often heroic initiative to locate IPE within an already crowded curriculum’ [56].

The statistically significant differences in evaluative responses between student professional groups are intriguing and we can only speculate on reasons. The pharmacy students were most positive. This might reflect their interest in the changing role of pharmacists in providing medication management services [57] along with the largely uniprofessional and campus-based curriculum pharmacy students had experienced to date; hence their enthusiasm to interact with students from other disciplines. It may also reflect the nature of the cases which all involved challenging, but solvable pharmacy problems (it proved easier to standardise the pharmacy complexity than other case aspects). The differences between medical students’ responses and other disciplines’ may reflect medical student focus on another (barrier) clinical assessment task due the same week and/or the junior level of the medical students compared with other students.

The contextual curriculum issues that limited engagement (assessment variation, timing, junior medical students) are all resolvable. It is not clear whether the novel video task, which challenged students, and was disliked by some, aided engagement and the achievement of IPL outcomes. There was surprisingly little rich comment overall about this novel creative assessment task which had been designed to require collaboration and engender emergence. While there may be ‘a fundamental tension between the engagement and level of achievement which can result from a creative open ended task and the constraints associated with assessment’ [58] some health students were hostile to the request for creativity and the video requirement may not have resulted in increased task engagement.

The anticipated IPL outcomes were demonstrated by the evaluation. The overarching themes of engagement and collaboration-in-action suggest that the IPL activity was relevant to preparation for future IPP. Students learnt about the contributions other professions could make and how their priorities varied. Collaboration-in-action was evident as students participated in negotiation and conflict resolution to resolve professional priorities into a patient-centered plan. Learning outcomes b and c both require development of new (emergent) collective knowledge. This was evidenced by the assessable products which were varied and creative (unpublished data) and by student self-report of their experience. Some students were able to provide a clear description of the synthesis that occurred as they worked together to produce their emergent products that addressed the patient’s needs. Our findings thus support the relevance of complexity theory for IPL and add detailed exemplar of a single IPL activity to the existing literature which is more programmatic in focus [37, 38].

### Limitations

The evaluation survey response rate of 26 % was low, probably due to the excessive gap between the completion of the HCC and the request to participate in the survey. Delays in marking the written abstracts, meant it took four weeks from event participation until students received their results and were asked to evaluate the experience. Nevertheless, student numbers overall were large and our range of qualitative comments suggests we captured both negative and positive experiences of the IPL activity.

### Next steps

Attempts to improve or remove the issues that limited engagement (assessment variation, timing, junior medical students) are being made for future iterations of the activity. We also consider that use of a peer feedback tool will incentivise participation. An investigation of students’ video production skills will be undertaken and supplemented by provision of more extensive educational support for video production. We will review the assessment rubrics used for the video and the written management plan to ensure that they promote emergence by adequately valuing diverse thinking (creativity) and evidence of integrated discipline knowledge (a *cohesive* patient-centred plan). Clear boundaries and ground rules give groups the security to take risks and be creative [54]. Finally, more work needs to be done, by us and others, to develop psychometrically valid and reliable methods of assessment for IPL activities. Without this, it is hard to obtain support for IPL activities to be given appropriate and significant assessment weighting within the UOS of individual disciplines.

Future research aims to study student teams *while* they are working to understand how and when their interactions most successfully create new collective knowledge. Similar research has been undertaken in another setting, where psychology students videoed student groups making health promotion videos [59]. A written-only task could be provided to some teams and their interactions compared with those of teams undertaking the video task. A reflexive session with participating student groups will then be undertaken and videoed (using the video-reflexive methodology [26]) so that a rich range of insights are available into successful and unsuccessful collaboration (and the contributions a creative video task makes). This would enable us to better support the IPL collaborative process in future iterations.

## Conclusion

We have demonstrated the feasibility and acceptability of a large-scale student-directed case-based IPL activity. Educational design (cases, logistics and assessment tasks) founded in complexity theory (encouraging emergence, diversity and self-organisation) resulted in demonstrable student engagement and collaboration-in action. Our findings provide support for the applicability of complexity theory for developing meaningful IPL activities for health professional students.

## Abbreviations

DR, diagnostic radiology student; EP, exercise physiology student; HCC, health collaboration challenge; IPE, interprofessional education; IPL, interprofessional learning; IPP –interprofessional practice; LMS, learning management system; M, medical student; N, nursing student; OT, occupational therapy student; P, pharmacy student; PT, physiotherapy student; SP, speech pathology; UOS, unit of study

## Additional file

Additional file 1: Sample Case Study: ‘Jane Murphy’. 530 word summary of the 1000 word case provided to students. (DOCX 122 kb)
