# Supplementary material for: Using complexity theory to develop a student-directed interprofessional learning activity for 1220 healthcare students
Source: BMC Med Educ. 2016 Aug 8;16:199. doi: 10.1186/s12909-016-0717-y (PMC4977619; doi:10.1186/s12909-016-0717-y)
Supplement: Additional file 1: — Sample Case Study: ‘Jane Murphy’. 530 word summary of the 1000 word case provided to students. (DOCX 122 kb) [file 12909_2016_717_MOESM1_ESM.docx]

**Supplementary File 1**

**Sample Case Study: ‘Jane Murphy’ ( 530 word summary of the 1000 word case provided to students)**

| Jane is a 59 year old who was diagnosed with bulbar onset amyotrophic lateral sclerosis in April 2014 and by July 2014 her condition had progressed significantly. She had weakness in all limbs, daily choking episodes, unintelligible speech and needed assistance with activities of daily living. She proceeded to have a percutaneous gastrostomy (PEG) and received assistive communication devices and mobility aids. Her tenacious saliva required a suctioning unit and a suprapubic catheter (SPC) was inserted for urinary frequency. Her truncal and neck weakness were causing pain in her neck and this was treated with physiotherapy and a cervical collar.  Jane can no longer take care of herself. She is unable to use her walker and needs a wheel chair. She needs assistance with all activities of daily living and is predicted to deteriorate quickly. Jane is also having problems with pain, breathing and emotional issues dealing with her rapidly deteriorating condition. Jane has agreed for admission to the palliative care unit to improve her quality of life including management of her breathlessness and pain. Jane is reluctant to take opioids she is worried about sedation and constipation. This admission is also to facilitate discharge planning to an aged care facility. Jane is not for resuscitation but wants all acute medical conditions treated if this would help her quality of life. Jane’s sister Mary is her next of kin and also holds enduring medical guardianship.  On admission, Jane is highly anxious about being able to communicate her needs. Jane uses her iPad to communicate but can use only one finger in her right hand which has poor control and strength and only when it is positioned in a very specific way. Jane blinks her eyes in response to YES and NO questions. Jane wants to have a shower every second day and needs a hoist. She wants to sit in a recliner chair during the day. Jane is PEG fed and is at aspiration risk. She has a SPC in situ which looks excoriated. Jane has contractures in her left upper limb. She has pain and weakness in her neck. She also has pain in her right knee. She needs help with breathing and sputum clearance. If her neck is not positioned she has breathing problems. She cannot mobilise and needs full assistance with transfers and self-care. She is at risk of pressure areas.  **Social Assessment**  Jane is single and worked as an office administrative assistant and played competitive netball. Jane lives with her mother in a rented apartment and was her mother’s carer. Jane has strong Catholic faith and is an active member of her parish.  **Medications**  Fentanyl transdermal patch 12 micrograms/hour 1 every three days  Hydromorphone 0.5mg PEG q2h PRN  Riluzole 50mg PEG daily  Sertraline 50mg PEG daily  Solifenacin 5mg PEG daily  Esomeprazole 40mg PEG daily  **Allergies -** Intolerance reported to morphine and diazepam but no true allergies  **Physical Examination** – provided to students  **Your task**  Although Jane‘s admission is for symptom management and discharge planning, this may be her terminal admission too. You are the health team who will take care of Jane during this palliative care inpatient admission. You are tasked with developing a 4-week comprehensive plan for Jane’s care. |
| --- |
